# ERAS including minimal Narcotic Pain Management Is Successful After Heart Transplant and LVAD Implantation: A Single Center Review

**DOI:** 10.1016/j.jhlto.2025.100267

**Published:** 2025-04-08

**Authors:** Joseph Elder, Jennifer McComb, David Blitzer, Seth Lirette, Kristin Lytal, John Morton, Asim Mohammed, Hannah Copeland

**Affiliations:** a Lutheran Hospital – Fort Wayne, Indiana; b Columbia University, Department of Surgery, Division of Cardiovascular Surgery, New York, NY; c Fulcrum, Jackson MI

**Keywords:** ERAS, Heart transplant, LVAD

## Abstract

**Objective:**

To assess the outcomes of an enhanced recovery after surgery (ERAS) protocol for heart failure patients receiving heart transplant or left ventricular assist device (LVAD) surgery.

**Methods:**

A single center, retrospective IRB study reviewed consecutive heart transplant and LVAD implantation patients from May 2020 to May 2022. Patients received an organized ERAS protocol. The following data points were evaluated: demographics, medication history, date of surgery, acute medication use, nutrition and bowel function assessment, time to liberate from mechanical ventilation, delirium screening, pain scores, functional status, time from implant to hospital discharge, and pain medications at both discharge and 30-day follow-up.

**Results:**

Thirty-two patients received ERAS: 18 heart transplants and 14 LVAD implants. One heart transplant was excluded from ERAS protocol due to delayed sternal closure for 72 hours after surgery. For a seven-day postoperative observation period, three patients (9%) had a positive delirium score. Pain scores were acceptable. The mean morphine milligram equivalent (MME) was 26.25 mg per day. No patient developed gastrointestinal complications. The average length of stay after surgery was 25 days for heart transplant, and 19 days for LVAD implant. No patients were prescribed opioids at discharge, and no patients reported opioid use over baseline at 30-days.

**Conclusion:**

For heart transplant and LVAD surgery, ERAS reduced opioid consumption, preserved mechanical ventilation time, and limited acute delirium. ERAS limited postoperative opioid induced gastroparesis while preserving acceptable pain scores, maintained the length of hospitalization, and prevented unnecessary opioid prescribing at discharge up to and including 30 days thereafter.

## Background

While the application of ERAS to cardiac surgery has grown, the potential impact in LVAD and heart transplant surgery is evolving. Across the surgical population broadly, ERAS has decreased postoperative complications, length of stay, and cost of care. ERAS increases overall patient satisfaction and cognitive recovery.^1^, ^2^ Implementation of ERAS in the LVAD and heart transplant surgery patient is more than simply the execution of a protocol, it contains core principles that together surpass the traditional surgical recovery by decreasing exposure to recovery practices carrying a higher risk of negative clinical consequence.

As a bundled approach to perioperative care, Enhanced Recovery After Surgery (ERAS) is implemented upon the established research that patients recover from surgery most optimally when physiologic, cognitive, and emotional stresses are countered with sustainable, and preferably more preventative measures, especially during the perioperative period. This includes upholding standards of care that have been established to prevent acute patient morbidity, and improve quality functional status beginning with the operative phase continuing into the months following surgery. The goal of ERAS is to decrease exposure to practice interventions that generally carry a higher risk of negative clinical consequence, such as longer time on the ventilator, longer time in the intensive care unit, and perhaps most prohibitively, potentially harmful opioid effects such as feeding intolerance, cognitive impairment, delirium, and prolonged medication dependence. Providing milder forms of perioperative pain management, for example, as one part of an ERAS model in heart transplant and LVAD surgery patients could potentially return the patient to baseline functional status more quickly after surgery.^2^ The hypothesis that surgical outcomes and patient satisfaction could be maintained, but also improved in specific areas, without potentially life altering cognitive impairment or opioid dependence, is a breakthrough for patients after LVAD or heart transplant surgery. ERAS programs have been developed for nearly every surgical subspecialty; while interest in the application of ERAS to cardiac surgery on the broad spectrum has grown, the specific potential impact in LVAD and heart transplant surgery is still evolving. Across the surgical patient population broadly, ERAS has demonstrated decreased postoperative complications, length of hospital stay, cost of care, while perhaps most importantly, increasing overall patient satisfaction.^2^, ^3^ Recognizing the improvements in other surgical settings – implementation of ERAS in the high acuity LVAD and heart transplant surgery patient is more than simply the execution of an individual institution protocol that has demonstrated efficacy in other surgery populations. ERAS in cardiac surgery programs requires a program culture shift that outwardly manifests at the bedside as an abundance of targeted, multidisciplinary orders focusing on core principles that when put together, surpass the traditional care model during and after heart transplant or LVAD surgery.^3^, ^4^ The goal is to describe the single institution’s ability to more definitively explore the outcomes of ERAS when applied to heart failure patients receiving LVAD and heart transplant surgery.

Fundamentally, ERAS involves a bundle of interventions applied to the patient across the continuum of perioperative surgical care, but the real challenge is building the culture and perception surrounding the perioperative and immediate postoperative care of surgery patients, and for the focus of this review, the program culture establishment around LVAD implant and heart transplant surgery.^3^, ^4^ By the nature of the cardiogenic failure, heart transplant and LVAD implant patients generally have a high surgical acuity. Overcoming traditional perioperative care models represents one of the many challenges to implementing ERAS in this surgical patient category.^5^, ^6^, ^7^ ERAS may be unfamiliar to a patient or their advocate, an established provider, or bedside caretaker and the hesitation to perform a detail in the ERAS bundle is typically evident as opposition (often due to unfamiliarity with ERAS). Initiating the ERAS bundle in a patient with advanced heart failure is a shared choice by the medical team and the patient.^6^, ^7^ Choosing to implement ERAS in one or a few patients is difficult in an unfamiliar institution; *sustaining* meaningful organizational change to apply it to the program, is even more challenging, due to familiarity and ease of the conventional care model that’s been practiced for decades. The lack of overall literature support to date that affirms successful ERAS performance in this specific patient cohort could further enhance the perioperative care of these patients.^8^, ^9^

The primary goal of the institution’s retrospective review is to assess the outcomes of an ERAS protocol in heart transplant and LVAD placement surgery for heart failure patients at a single transplant center. By establishing, documenting, and reporting outcomes on an ERAS model that transcends the typical heart transplant and LVAD surgery recovery with a more patient-centered system of care to augment completeness of recovery - the goal of sharing the responsibility to improve care for heart failure patients is realized. The review details the single center’s ERAS protocol in the heart transplant and LVAD surgery program, focusing on both successful clinical and functional outcomes; demonstrating a safe, efficacious, and clinically significant use of ERAS in the heart transplant and LVAD surgery patient population.

## Methods

A retrospective, IRB approved single transplant center chart review of consecutive heart transplant and LVAD patients from May 2020 to May 2022 who received an organized, facility approved ERAS protocol. Definitive elements of the ERAS protocol will be described in greater detail in the discussion section. The perioperative medication portion of the individual program’s protocol, a major focus of ERAS, included scheduled acetaminophen, tramadol, gabapentin, and lidocaine patches immediately following surgery. Additionally, acetaminophen 650 milligrams and gabapentin 300 milligrams by mouth were provided to each patient in a single preoperative dose. Postoperative dosing of medication was adjusted as needed, based on end organ function of the patient's kidneys (e.g. urine output, blood urea nitrogen, serum creatinine) and liver (e.g. AST, ALT, Total bilirubin). The postoperative medication portion of the protocol, including dose adjustments and parameters in which adjustments were based on end organ function, is included in Table 1.

**Table 1:** ERAS Medication Protocol Table

Medication	Dose	Adjustment (s) as Needed	Monitoring	Adjusted Dose (s)
Acetaminophen	650 mg per NG/OG every 6 h	liver insufficiency	BPS/NRS LFTs, Total Bilirubin	650 mg per NG/OG every 8 h
Tramadol	50 mg per NG/OG every 6 h	kidney or liver insufficiency	BPS/NRS Urine Output BUN/SCr LFTs, Total Bilirubin	50 mg per NG/OG every 8−12 h
Gabapentin	200 mg per NG/OG every 8 h	kidney insufficiency	BPS/NRS Urine Output BUN/SCr	100 mg per NG/OG every 8 h
Lidocaine Patch	4% patch to chest wall 12 h on 12 h off	N/A	BPS/NRS Skin irritation / rash	N/A

*kidney insufficiency - defined as urine output less than 0.5 ml/kg/hr for 8 h OR BUN/SCr increase by 40% or more within 24 h

*liver insufficiency - defined as AST/ALT or total bilirubin increase above 5x the upper limit of normal

For this review, the following data points were evaluated: baseline patient demographics, perioperative laboratory data, baseline and acutely prescribed inpatient medications (*including* opioid medications), functional status, date of surgery, and date of hospital discharge. Additionally, postoperative administration of opioids, time to extubate, delirium screening, objective pain assessment, nutritional status, and pain medications prescribed at both discharge and thirty-day outpatient follow-up appointment were evaluated.

Objective assessments described in focused areas were completed by bedside nursing, dieticians, clinical pharmacists, and providers. Postoperative administration of opioids was evaluated over each twenty-four-hour period using review of medications administered, and converting the total amount given (in micrograms or milligrams) to *morphine milligram equivalents per day* (MMEs). Table 2 describes the MMEs of typical opioid use in perioperative care compared to that of tramadol, which is one medication used in the institution's ERAS protocol. Postoperative objective delirium screening was completed once per eight-hour shift using the *Confusion Assessment Method for the ICU* (CAM-ICU), which is a standardized, internationally accepted method supported by the *Pain, Agitation, Delirium, Immobility, and Sleep Disruption (PADIS)* guidelines of medical and surgical intensive care patients. The CAM-ICU includes evaluation of four domains: acute change or fluctuating course of mental status, inattention, altered level of consciousness, and disorganized thinking.^10^ Postoperative pain assessment was completed using the *behavioral pain scale* (BPS) score where applicable for mechanically ventilated patients, and the *numerical rating scale* (NRS) in non-mechanically ventilated, communicative patients. Where applicable for a given patient, the pain assessment was completed once every four hours, and thirty minutes after administration of any medication dose used as needed for pain. The BPS consists of 3 domains: facial expression, upper limb movement, and compliance with mechanical ventilation. The domains are scored, and are additive (BPS score range from 3–12) with the acceptable score being less than six. The NRS consists of a numerical rating scale, zero being no pain, ten being the worst possible pain, with the acceptable score being less than four.^10^

**Table 2:** Opioid Conversion Table (Morphine Milligram Equivalents).

Opioid	mg/day	Conversion Factor	Morphine Milligram Equivalents (MME)	Example mg/24 h Period
Fentanyl	0.6	100	60	Fentanyl 0.05 mg every 2 hr / 24 h
Hydrocodone	20	1	20	Hydrocodone 5 mg every 6 hr / 24 h
Hydromorphone	8	4	32	Hydromorphone 1 mg every 3 hr / 24 h
Morphine	24	1	24	Morphine 2 mg every 2 hrs / 24 h
Oxycodone	20	1.5	30	Oxycodone 5 mg every 6 hrs / 24 h
Tramadol	200	0.1	20	Tramadol 50 mg every 6 hrs / 24 h

The BPS and NRS are internationally acceptable intensive care pain assessments promoted by the *Society of Critical Care Medicine through the PADIS guidelines* and the *ICU Liberation Bundle* (the ABCDEF Bundle). The ABCDEF bundle has proven lifesaving benefits for both medical and surgical ICU patients. When implementing ERAS, many of the core elements are focused congruently to the principles of the ABCDEF Bundle and the PADIS guidelines, the most fundamental congruence being truly *re-humanizing* care across the intensive care population.^10^, ^11^

## Results

Thirty-two patients received ERAS: eighteen heart transplants and fourteen LVAD implants. The mean patient age was 60 years (SD: 48–73) and the majority of the patients (80%) were male. Nine patients (28%) were taking historically prescribed opioid medications at baseline prior to surgery.

To apply acuity characteristics to the patient population, the waitlist status of the eighteen heart transplant patients in order of severity: Impella with sustained ventricular arrhythmia status 1 (n=1), VA ECMO status 1 (n=1), Impella status two (n = 8), intra-aortic balloon pump status two (n = 2), LVAD with complication or deterioration (e.g. driveline infection) status three (n=4), and LVAD without complication (e.g. driveline infection) status four (n=2). The INTERMACS profiles of the fourteen LVAD patients were primarily INTERMACS profile two (n=8), followed by INTERMACS profile three (n=3), INTERMACS profile one (n=2), and finally INTERMACS profile 4 (n=1).

One heart transplant patient was excluded from the ERAS protocol for seventy-two hours after heart transplant surgery due to delayed sternal closure secondary to bleeding. No patients received regional intercostal or other nerve blocks as part of the multi-modal ERAS approach. No patient developed gastrointestinal complications (e.g. vomiting or ileus). All patients were on inhaled nitric oxide (iNO) per heart transplant/LVAD protocol in addition to ERAS following surgery to manage right ventricular (RV) hemodynamics, titrated to normal RV function and pulmonary artery systolic pressure. After iNO was weaned over an average of thirty-six hours postoperatively, the patients were weaned to extubate. For venous thromboembolism (VTE) prophylaxis, thirty-two patients (100%) were provided mechanical prophylaxis with bilateral lower extremity sequential compression devices, twenty patients (60%) had dose appropriate chemical prophylaxis with subcutaneous heparin, or low intensity heparin infusion (aPTT 1.5–2 times control) in place within 24 hours of surgery. All patients (100%) had chemical prophylaxis in place with either subcutaneous heparin, or low intensity heparin infusion within 48 hours of surgery. Fourteen LVAD implant patients (100%) received low intensity heparin infusion following implant surgery due to the precise coagulation balance clinically necessary, and all LVAD implant patients were converted to oral vitamin K antagonist therapy at discharge. Five heart transplants (28%) received low intensity heparin infusion due to a transient indication for anticoagulation (e.g. isolated history of VTE or hemoglobin drift with higher transfusion threshold), however no heart transplant patients required continued anticoagulation beyond the inpatient setting. No patients required intermediate or high intensity anticoagulation for an acute indication such as new atrial fibrillation or thrombosis, and no patients developed venous thromboembolism. For a seven-day postoperative observation period, three patients (9%) had a CAM-ICU positive assessment suggesting presence of acute delirium, however only one patient (3%) had more than three positive daily assessments, and no patients had isolated hyperactive delirium recorded (e.g. hallucinations). BPS scores were consistently five or less, with the internationally acceptable goal being BPS score less than six. NRS scores were consistently six or less (moderate pain). The mean morphine milligram equivalent (MME) used was 26.25 mg per day, 20 mg per day being attributed to the ERAS protocol. The primary MME was from scheduled tramadol 50 milligrams by mouth every six hours, which was dose adjusted as needed for kidney and liver dysfunction (included in Table 1). The average length of stay after surgery was 25 days for heart transplant, and 19 days for LVAD implant. No patients were prescribed narcotic analgesics at discharge, and no patients reported narcotic use over baseline at 30-day outpatient follow-up.

For heart transplant and LVAD surgery, the ERAS protocol with reduced opioid consumption prevented the incidence of acute postoperative opioid induced gastroparesis or ileus, preserved invasive mechanical respiratory support time, and limited ICU-delirium compared to routine conventional postoperative care (including opioid use), while preserving acceptable pain scores, preserving the length of hospitalization, and preventing unnecessary opioid prescribing at discharge up to and including 30 day follow up appointment.

## Discussion

A primary limitation is the single center, retrospective review design of the study. In addition, due to the small sample size, it is difficult to discern external validity despite the benefits of ERAS described in the results, particularly related to MMEs utilized, avoidance of acquired delirium leading to prolonged mechanical ventilation, and opioid prescribing at discharge over baseline opioid medication use.

The essential medication elements of the facility approved ERAS protocol for heart transplant and LVAD implantation does not differ from ERAS protocols of other surgical subspecialty programs, including those described in the literature for cardiac surgery. It includes an opioid sparing analgesia strategy, at this institution: scheduled acetaminophen, tramadol, gabapentin, and topical lidocaine. The dosing was adjusted as needed, based on end organ function as described in the methods. Acetaminophen, tramadol, gabapentin, and lidocaine are important medications in the institution's ERAS protocol, requiring daily clinical pharmacist review and dose adjustment based on kidney and liver function. Gabapentin dosing was limited to a max of 600 milligrams per day for creatinine clearance less than 30 milliliters per minute; tramadol dosing was limited to a max of 200 milligrams per day for creatinine clearance less than 30 milliliters per minute and 100 milligrams per day for moderate-severe hepatic impairment (total bilirubin above 2 milligrams per deciliter, and or aminotransferase above five times upper limit of normal). No patients received regional (e.g. intercostal bupivacaine) nerve block as part of the multi-modal ERAS approach, as early denervated hearts (e.g. heart transplant) may not reflexively compensate adequately for the hemodynamic lability introduced with regional anesthetic use.

By mitigating the risk associated with unilaterally providing narcotic analgesia peri and postoperatively, more muted medications at lower doses as part of an ERAS model increase the opportunity for patients to consciously participate in their breathing and mobility, allowing them to more quickly recover. Avoiding collateral opioid effects, especially with continuous infusion or continued intermittent dosing, promotes respiratory effort, hemodynamic stability, nutritional tolerance, early mobility, and cognitive restoration. Further narrowing the clinical role opioid infusions have during and after heart transplant and LVAD implant surgery, diminishes the need for prolonged vasoactive and mechanical ventilatory support. The results of this review affirm this research applicability to heart transplant and LVAD surgery.

The acute medication bundle is important, however, other essential elements are included in the center’s heart transplant and LVAD surgery ERAS bundle: nutrition assessment focused on prehabilitation with optimization of hemoglobin A1C, serum albumin and prealbumin, in addition to early and adequate enteral nutrition to promote surgical site healing and re-engagement of musculoskeletal and diaphragm conditioning; surgical site infection reduction and sternal closure bundle with targeted perioperative antibiotics (and re-dosing of perioperative antibiotics for surgery lasting longer than 4 hours), standardized topical cleansing, and maintenance of surgical field sterility; glycemic control focused on avoidance of hypoglycemia to enhance cognitive function, in addition to reliance on insulin support for stress hyperglycemia to promote healing and limit postoperative infection risk; avoidance of postoperative core body hypothermia (less than 34 degrees Celsius) or hyperthermia (greater than 37.5 degrees Celsius); maintenance of chest tube patency with close output monitoring; early detection of kidney stress with hourly urine output monitoring and avoidance of agents or events that promote kidney injury (e.g. non-steroidal anti-inflammatory medications, intravenous contrast, angiotensin blocking medications, hypovolemia).

The ERAS protocol consists of early goal directed fluid management focused on blood component stewardship, advanced cardiopulmonary hemodynamic monitoring, and peripheral blood measurement of oxygen carrying and coagulation status (e.g. complete blood count, thromboelastogram, and fibrinogen) in addition to enzymatic coagulation measurements (e.g. activated clotting time, partial thromboplastin time, and prothrombin time) to promote targeted component based blood product transfusion. The ERAS patients' transfusion rates aligned favorably with the transfusion rates described in the literature (specifically transfusion of packed red blood cells and fresh frozen plasma). While not a direct comparison, a recent meta-analysis’ data reveals a trend toward less products used in the institutions ERAS patients (overall fresh frozen plasma transfusion rate of 0 [IQR: 0–3 U] and overall red blood cell transfusion rate of 4 units [IQR: 2–5 U] compared to available literature 3–14 U.^12^ If a true trend of less transfused product is realized, it is a benefit to both the patient (e.g. decreasing risk for volume overload, right ventricular heart strain, and antigenic load) and to the tertiary health center (e.g. promoting blood component stewardship and reducing overall care expenditure cost).

Patients were screened for and protected against the operative risk of venous thromboembolism. Mechanical venous thromboembolism prophylaxis was applied to bilateral lower extremities with sequential compression devices throughout the hospital stay, in addition to chemical thromboembolism prophylaxis before, and as soon as possible after surgery when hemostasis was maintained (e.g. chest tube output less than thirty milliliters per hour) typically within twenty-four, but absolutely within forty-eight hours. For chemical thromboembolism prophylaxis, subcutaneous unfractionated heparin (UFH) was provided with 5000 units subcutaneously every eight hours. For patients with elevated body mass index greater than forty kilograms per meter squared, the UFH dose was increased to 7500 units subcutaneously every eight hours. For patients with an overt indication for continued anticoagulation (e.g. LVAD implantation), UFH infusion to a targeted low intensity therapeutic range (e.g. aPTT 1.5–2x control) was utilized until oral vitamin K antagonist was safely therapeutic. All patients had chemical prophylaxis in place with either subcutaneous heparin, or low intensity heparin infusion within 48 hours of surgery.

Optimization of nutrition in both the pre and post-operative periods is essential to healing after surgery. Patients were enterally fed as soon as possible after surgery typically within twenty-four hours, and were monitored closely for tolerance and optimization by nursing with daily clinical dietician and pharmacist evaluation. Early postoperative feeding tolerance was improved in these patients compared to traditional post-surgical care, due to the opioid sparing medication regimen applied with ERAS. No patients in the program developed postoperative nausea or vomiting, and no patients developed abdominal distention related to a higher than desired gastric residual volume. Enteral feeding tolerance by clinical bedside assessment was affirmed by optimizing serum electrolytes, avoiding intermittent or continuous opioids, and only checking gastric residual volume when clinically significant gastroparesis is suspected (e.g. new nausea or vomiting with abdominal distention). No patients developed paralytic ileus requiring medical intervention including abdominal computed tomography evaluation, surgery, or systemic partial mu-opioid receptor antagonism (e.g. methylnaltrexone) to promote return of bowel function. The overall nutrition bundle supports the patient’s recovery in addition to lowering cost to the health system by preventing the described complications that lead to added cost, patient morbidity, and length of stay.

Early mobility and exercise (e.g. daily physical, occupational, and speech therapy evaluation and treatment) facilitated cognitive restoration, positively impacting the day-night cycle (e.g. limit REM sleep disruption), and further decrease the chances of acute delirium as described in the PADIS guidelines. In the institution's ERAS protocol, therapists in each discipline utilize their skills to take the smallest early interventions (e.g. passive range of motion) and build on them to more quickly get the patient to full participation with all their normal activities.

Prior to implementing the ERAS protocol, the institution had a portion of LVAD patients successfully implanted (n=11) over a 24 month period that were reviewed. While it is difficult to make a comparison with small sample size using a single institutions data (n=11 pre ERAS and n=32 post ERAS), the pre ERAS group revealed inferior data points with MME being 78 mg per day (ERAS MME 26.25 mg per day), length of stay after implant surgery being 20.5 days (ERAS LOS 19 days), and finally, forty five percent of the patients receiving opioid prescriptions at discharge over baseline (ERAS opioid prescribing zero percent over baseline).

Traditional postoperative care including scheduled or intermittent opioid medications in addition to deviations from the practices previously described from the institution's ERAS protocol, have increasingly been implicated in the development of acute agitation, sleep cycle impairment, immobility, and delirium. Acute ICU delirium is one of the costliest, both to the health system and to the patient; deleterious to the system by way of increased time on full mechanical and vasoactive support and greater time in the ICU. The patient is exposed to potentially less predictable interventions, that by themselves may not be inherently harmful but when put together may carry increased risk of cognitive and physical impairment, post-traumatic stress disorder, and mortality. While it is recognized that the two surgeries described (heart transplant and LVAD implant) generally carry a lower risk of harm overall, the goal of ERAS in these two surgeries is ultimately to be a singular organized improvement that can at least provide the same quality and efficacy, and at a minimum prevent prolonged opioid prescribing, and a bad outcome for a patient.^9^

In addition to the institution's results, the primary outcome achieved was the adoption of a culture changing surgery care model focused on preserving and returning the patient to their physical, cognitive, and overall functional status. Future research should further define the amount of investment in time and resources an aspiring institution should devote to each individual element of the ERAS bundle when applying it to heart transplant and LVAD implantation. In addition, this research should promote the development of institutional toolkits that can be used to develop individual programs and apply the elements described to patients. ERAS in heart transplant and LVAD implantation focuses on whole human recovery, promoting physiologic and cognitive restoration, limiting the risk of delirium and universally: returning patients to normal baseline functional status after surgery.

## Ethics Statement

This study received approval from the Institutional Review Board; project approval 09/15/22.

## Disclosures

Hannah Copeland has been a speaker for Paragonix and Abbott. The authors have no other disclosures to report.
